# Genome-wide identification of YABBY genes in three *Cymbidium* species and expression patterns in *C. ensifolium* (Orchidaceae)

**DOI:** 10.3389/fpls.2022.995734

**Published:** 2022-11-24

**Authors:** Qian-Qian Wang, Yuan-Yuan Li, Jiating Chen, Meng-Jia Zhu, Xuedie Liu, Zhuang Zhou, Diyang Zhang, Zhong-Jian Liu, Siren Lan

**Affiliations:** ^1^ Key Laboratory of National Forestry and Grassland Administration for Orchid Conservation and Utilization at College of Landscape Architecture, Fujian Agriculture and Forestry University, Fuzhou, China; ^2^ College of Forestry, Fujian Agriculture and Forestry University, Fuzhou, China; ^3^ Zhejiang Institute of Subtropical Crops, Zhejiang Academy of Agricultural Sciences, Wenzhou, China

**Keywords:** YABBY genes, Orchidaceae, *Cymbidium*, expression pattern, genome-wide

## Abstract

Members of the YABBY gene family play significant roles in lamina development in cotyledons, floral organs, and other lateral organs. The Orchidaceae family is one of the largest angiosperm groups. Some YABBYs have been reported in Orchidaceae. However, the function of YABBY genes in *Cymbidium* is currently unknown. In this study, 24 YABBY genes were identified in *Cymbidium ensifolium*, *C. goeringii*, and *C. sinense.* We analyzed the conserved domains and motifs, the phylogenetic relationships, chromosome distribution, collinear correlation, and *cis*-elements of these three species. We also analyzed expression patterns of *C. ensifolium* and *C. goeringii*. Phylogenetic relationships analysis indicated that 24 YABBY genes were clustered in four groups, INO, CRC/DL, YAB2, and YAB3/FIL. For most YABBY genes, the zinc finger domain was located near the N-terminus and the helix-loop-helix domain (YABBY domain) near the C-terminus. Chromosomal location analysis results suggested that only *C. goeringii* YABBY has tandem repeat genes. Almost all the YABBY genes displayed corresponding one-to-one relationships in the syntenic relationships analysis. *Cis*-elements analysis indicated that most elements were clustered in light-responsive elements, followed by MeJA-responsive elements. Expression patterns showed that YAB2 genes have high expression in floral organs. RT-qPCR analysis showed high expression of *CeYAB3* in lip, petal, and in the gynostemium. *CeCRC* and *CeYAB2.2* were highly expressed in gynostemium. These findings provide valuable information of YABBY genes in *Cymbidium* species and the function in Orchidaceae.

## Introduction

The seed plant-specific YABBY gene family, belonging to the zinc-finger superfamily, plays significant roles in lamina development in cotyledons, floral organs, and outer ovule integuments (Finet et al., 2016). YABBY genes encode transcription factors which contain two domains: a zinc finger domain located near the N-terminus and a helix-loop-helix domain (YABBY domain) located near the C-terminus (Bowman and Smyth, 1999). Six genes have been identified in *Arabidopsis thaliana*, and were clustered into five subfamilies: FIL/YAB3, CRC, INO, YAB2, and YAB5 (Siegfried et al., 1999). FIL, YAB2, YAB3, and YAB5 are expressed in leaf and floral organs and have been termed ‘vegetative YABBYs’. CRC and INO are essential in developing carpels and ovules, respectively, and have been termed ‘reproductive YABBYs’ (Bowman and Smyth, 1999; Siegfried et al., 1999; Villanueva et al., 1999; Bartholmes et al., 2012; Soundararajan et al., 2019).

According to previous studies from expression characterization in *Arabidopsis* YABBY genes, FIL, YAB2 and YAB3 play essential roles in lateral organ development (Siegfried et al., 1999; Rudall and Bateman, 2002; Lora et al., 2011). CRC is restricted to carpels and nectaries in angiosperms (Siegfried et al., 1999). INO functions in the development of the outer integument of the ovule to the seed coat in *Arabidopsis*, and INO expresses in eudicots, eumagnoliids, and some basal angiosperms (Bowman, 2000; Yamada et al., 2003; McAbee et al., 2005; Lora et al., 2011; Yamada et al., 2011).

The genome-wide YABBY gene family has been identified in *Averrhoa carambola* (star fruit), *Cucumis sativus* (cucumber), *Lycopersicon esculentum* (tomato), *Oryza sativa* (rice), *Triticum aestivum* (wheat) and *Vitis vinifera* (grape) (Toriba et al., 2007; Han et al., 2015; Zhang et al., 2019; Hao et al., 2022; Li et al., 2022; Yin et al., 2022). In monocot plants, YABBY genes show functional divergence and are crucial for vegetative and reproductive development. For example, the YAB3 clade genes *ZYB9* and *ZYB14* play essential roles in flower development and regulate lateral outgrowth (Juarez et al., 2004). *OsDL*, a member of the CRC subfamily in *O. sativa*, is necessary for the development of the leaf midrib and the flower carpel specification (Nagasawa et al., 2003; Yamaguchi et al., 2004; Ohmori et al., 2008; Zhang et al., 2020). *OsYAB1*, belonging to the YAB2 clade, is mainly expressed in the primordia of the carpel and stamen (Jang et al., 2004). The *OsYAB3* gene may be necessary for the development of lateral organs and the growth and differentiation of leaf cells (Jang et al., 2004).

With an estimated > 28000 species, the Orchidaceae family is one of the largest angiosperm groups (Christenhusz and Byng, 2016). There are five subfamilies of Orchidaceae: Apostasioideae, Cypripedioideae, Vanilloideae, Orchidoideae, and Epidendroideae (Chase et al., 2003). The Orchidaceae show considerable diversity in epiphytic and terrestrial life forms and show unique flower morphologies and reproductive biology (Hsiao et al., 2011). Orchidaceae flowers show a variety of reliable floral morphological synapomorphies, such as a gynostemium (a fused structure of the pistils and stamens), a highly evolved petal termed labellum, and flowers with pollinia (Chase et al., 2003; Tsai et al., 2004). In the Orchidaceae family, genome-wide identification and expression patterns of YABBY genes were analyzed in *Apostasia shenzhenica* (Apostasioideae), *Dendrobium catenatum* (Epidendroideae), *Gastrodia elata* (Epidendroideae), and *Phalaenopsis equestris* (Epidendroideae) (Chen et al., 2020). However, studies of YABBY genes in the orchid tribe Cymbideae are still limited. *Cymbidium* is one of the most significant orchid genera for ornamental value because of its beautiful flowers (Ramya et al., 2019). Given the considerable role of YABBY genes in both vegetative and reproductive development, the identification of *Cymbidium ensifolium*, *C. goeringii*, and *C. sinense* will be employed, and the expression patterns of *C. ensifolium* will be analyzed in this study. This study provides new insights into the roles of YABBY genes and their contribution to the development of flower morphologies in *Cymbidium* subfamily of Orchidaceae.

## Methods

### Identification of YABBY genes from three *Cymbidium* species

The YABBY domain (PF04690) from PFAM was used as a query to search the protein database (El-Gebali et al., 2019). The genomes from *Cymbidium ensifolium*, *C. goeringii*, and *C. sinense* can be downloaded from their whole-genome sequencing data (Sun et al., 2021; Yang et al., 2021; Ai et al., 2021). HMM analysis (built in Tbtools) was used at an e value of 10^-5^ (Chen et al., 2018). BLASTP (https://blast.ncbi.nlm.nih.gov/Blast.cgi) was also used to search the protein database using *A. thaliana*’s YABBY sequences, which can be downloaded in the TAIR database (https://www.arabidopsis.org). Then, the CDD website (https://www.ncbi.nlm.nih.gov/Structure/bwrpsb/bwrpsb.cgi) was used to confirm the retrieved putative sequences. The aliphatic index (AI), grand average of hydrophobicity (GRAVY), instability index (II), and isoelectric points (pI) of the YABBY proteins were predicted using the ExPASy website (https://www.expasy.org/; Artimo et al., 2012). AtSubP (http://bioinfo3.noble.org/AtSubP/) was used to predict the subcellular localization of YABBY genes (Kaundal et al., 2010). The secondary structure was predicted using the SOPMA (https://npsa-prabi.ibcp.fr/cgi-bin/npsa_automat.pl?page=npsa_sopma.html) program (Rozewicki et al., 2019).

### Phylogenetic relationship analysis of YABBY genes

The TAIR database (https://www.arabidopsis.org/) was used to download the protein sequences of *Arabidopsis thaliana*. The sequences of *Oryza sativa*, *Phalaenopsis equestris*, *V. vinifera*, and *Zea mays* were downloaded from the NCBI website (https://www.ncbi.nlm.nih.gov/genbank/). The protein sequences of YABBY genes from *C. ensifolium*, *C. goeringii*, and *C. sinense* can be downloaded from their whole-genome sequencing data (Ai et al., 2021; Sun et al., 2021; Yang et al., 2021). Multiple alignments were carried out using the program MAFFT (Rozewicki et al., 2019). Maximum likelihood (ML) tree inference was carried out using RAxML (RAxML-HPC2 on XSEDE; Miller et al., 2011), and was under a GTRGAMMA substitution model with 1,000 bootstraps. The EVOLVIEW website (https://evolgenius.info/) was used for layouting the phylogenetic tree (He et al., 2016).

### Motifs of YABBY proteins and sequence alignment in three *Cymbidium* species

Conserved domains of YABBY genes were analyzed using the CDD website (https://www.ncbi.nlm.nih.gov/Structure/bwrpsb/bwrpsb.cgi), and motifs were analyzed using the default parameters of the MEME website (http://meme-suite.org/) (Artimo et al., 2012). Fifteen motifs were identified in this study. To investigate the YABBY domains and C2C2 zinc-finger domain, the WEBLOGO tool (built in Tbtools) was employed. Multiple sequence alignments were carried out using MAFFT (Rozewicki et al., 2019).

### Chromosome distribution and collinear correlation in three *Cymbidium* species

To analyze the chromosomal location of YABBY genes in three *Cymbidium* species, the Tbtools software was used to create gene distribution maps by uploading the YABBY sequence (Chen et al., 2018). To analyze syntenic relationships, one step MCScanx (built in Tbtools) was used to analyze YABBY genes of *C. ensifolium*, *C. goeringii*, and *C. sinense* (Chen et al., 2018).

### Promoter element analysis of YABBY genes in *C. ensifolium*, *C. goeringii*, and *C. sinense*

The 2000 bp regions upstream of the YABBY genes in *C. ensifolium*, *C. goeringii*, and *C. sinense* were extracted by TBTOOLS (Chen et al., 2018). Then, the *cis*-acting elements were identified by the PlantCare website (http://bioinformatics.psb.ugent.be/webtools/plantcare/html/; Zhang et al., 2018).

### RNA extraction and RT–qPCR analysis

Flower organs (petal, lip, and gynostemium) and leaves of *C. ensifolium* were collected, frozen in liquid nitrogen, and stored at 80°C until use. Total RNA was extracted using the Biospin Plant Total RNA Extraction Kit (Bioer Technology, Hangzhou, China). TransScript^®^ All-in-One First-Strand cDNA Synthesis SuperMix for qPCR (TransGen Biotech, Beijing, China) was used to create first-strand DNA and remove genomic DNA. The reaction conditions were 30 s at 94 °C and 45 cycles of 5 s at 94°C and 30 s at 60°C. Primers for the RT–qPCR analysis were designed by Primer Premier 5 software. GAPDH (JL008987) was used for normalization. Three biological replicates were performed in this study, and the expression data were quantified *via* the 2-△△CT method (Livak and Schmittgen, 2001).

## Results

### YABBY gene identification and sequence analysis in three *Cymbidium* species

Seven YABBY genes were found in *C. ensifolium*, nine in *C. goeringii*, and eight in *C. sinense*. The deduced protein length of YABBY genes ranged from 63 to 243 amino acids. The theoretical isoelectric point (pI) ranged from 6.11 to 10.75, and instability index (II) ranged from 32.78 to 57.09. The deducted grand average of hydrophilic values (GRAVY) of YABBY genes ranged from -1.155 to -0.232, and we found all the YABBY proteins were hydrophilic. The molecular weight (Mw) ranged from 7744.05 to 27185.43, and the aliphatic index (AI) ranged from 52.92 to 83.98 (**Table 1**). Subcellular localization results showed that all the YABBY genes were located in the nucleus, indicating that the nucleus may be where the YABBY genes function (**Supplementary Table S1**; Kaundal et al., 2010). The results of secondary structure prediction revealed that the average of α-helices, extended strands, β-turns, and random coils comprised 27.61, 14.13, 5.65, and 52.6% of the structure, respectively (**Supplementary Table S2**; Geourjon and Deléage, 1995).

**Table 1:** A list of YABBY genes in three *Cymbidium* species.

Gene ID^1^	Name	AA^2^(aa)	pI^3^	Mw^4^(kDa)	AI^5^	II^6^	GRAVY^7^	Clade^8^	Localizetion^9^
*JL015423*	*CeCRC*	194	9.38	21643.69	59.38	45.46	-0.565	CRC	Nucleus
*JL011339*	*CeYAB2.1*	181	8.5	19855.55	83.98	43.57	-0.299	YAB2	Nucleus
*JL000262*	*CeYAB2.2*	181	7.74	19949.4	73.81	51.69	-0.401	YAB2	Nucleus
*JL008521*	*CeYAB3.1*	221	6.79	24350.89	79.5	57.09	-0.235	YAB2	Nucleus
*JL005041*	*CeYAB3.2*	221	7.7	24671.23	75.48	44.98	-0.329	YAB3	Nucleus
*JL005324*	*CeYAB2.3*	185	8.45	20710.15	66.43	47.37	-0.612	YAB2	Nucleus
*JL012731*	*CeINO*	157	9.32	17921.56	67.77	41.43	-0.543	INO	Nucleus
*GL09549*	*CgCRC.1*	188	9.11	21386.4	59.89	32.78	-0.625	CRC	Nucleus
*GL08212*	*CgCRC.2*	193	9.38	21643.69	59.38	45.46	-0.565	CRC	Nucleus
*GL09374*	*CgYAB3*	220	7.7	24698.26	75.48	44.98	-0.342	YAB3	Nucleus
*GL12804*	*CgYAB2.1*	242	9.73	27003.06	78.23	36.7	-0.408	YAB2	Nucleus
*GL19435*	*CgYAB2.2*	184	8.55	20658.15	64.32	46.16	-0.621	YAB2	Nucleus
*GL30075*	*CgYAB2.3*	78	9.8	8941.14	53.29	36.67	-0.995	YAB2	Nucleus
*GL30077*	*CgYAB2.4*	143	8.84	16732.82	52.92	47.9	-0.873	YAB2	Nucleus
*GL30076*	*CgYAB2.5*	63	10.75	7744.05	58.62	47.3	-1.155	YAB2	Nucleus
*GL10103*	*CgYAB2.6*	70	8.69	8099.94	64.79	33.43	-0.793	YAB2	Nucleus
*Mol018025*	*CsCRC.1*	243	9.1	27185.43	76.71	49.97	-0.27	CRC	Nucleus
*Mol010228*	*CsCRC.2*	194	9.38	21643.69	59.38	45.46	-0.565	CRC	Nucleus
*Mol006632*	*CsYAB2.1*	181	8.19	19887.51	81.82	41.79	-0.346	YAB2	Nucleus
*Mol000581*	*CsYAB2.2*	181	8.58	19968.49	73.81	52.75	-0.406	YAB2	Nucleus
*Mol007225*	*CsYAB3.1*	220	7.15	24195.67	79.86	54.95	-0.232	YAB2	Nucleus
*Mol011195*	*CsYAB2.3*	185	8.45	20710.15	66.43	47.37	-0.612	YAB2	Nucleus
*Mol003404*	*CsYAB3.2*	161	7.96	18198.82	73.42	43.89	-0.569	YAB3	Nucleus
*Mol004846*	*CsINO*	184	6.11	20622.28	69.46	41.53	-0.561	INO	Nucleus

^1^Gene ID is annotated in the genome; ^2^AA, amino acid; ^3^pI, theoretical isoelectric point; ^4^Mw, molecular weight; ^5^AI, aliphatic index; ^6^II, instability index; ^7^GRAVY, the grand average of hydrophobicity; ^8^Clade is dependent on phylogenetic analysis, ^9^Localization, predicted by AtSubP (Kaundal et al., 2010). Raw data are listed in **Supplementary Tables S1** and **S2**.

### Phylogenetic relationship analysis of YABBY genes

**Figure 1:**
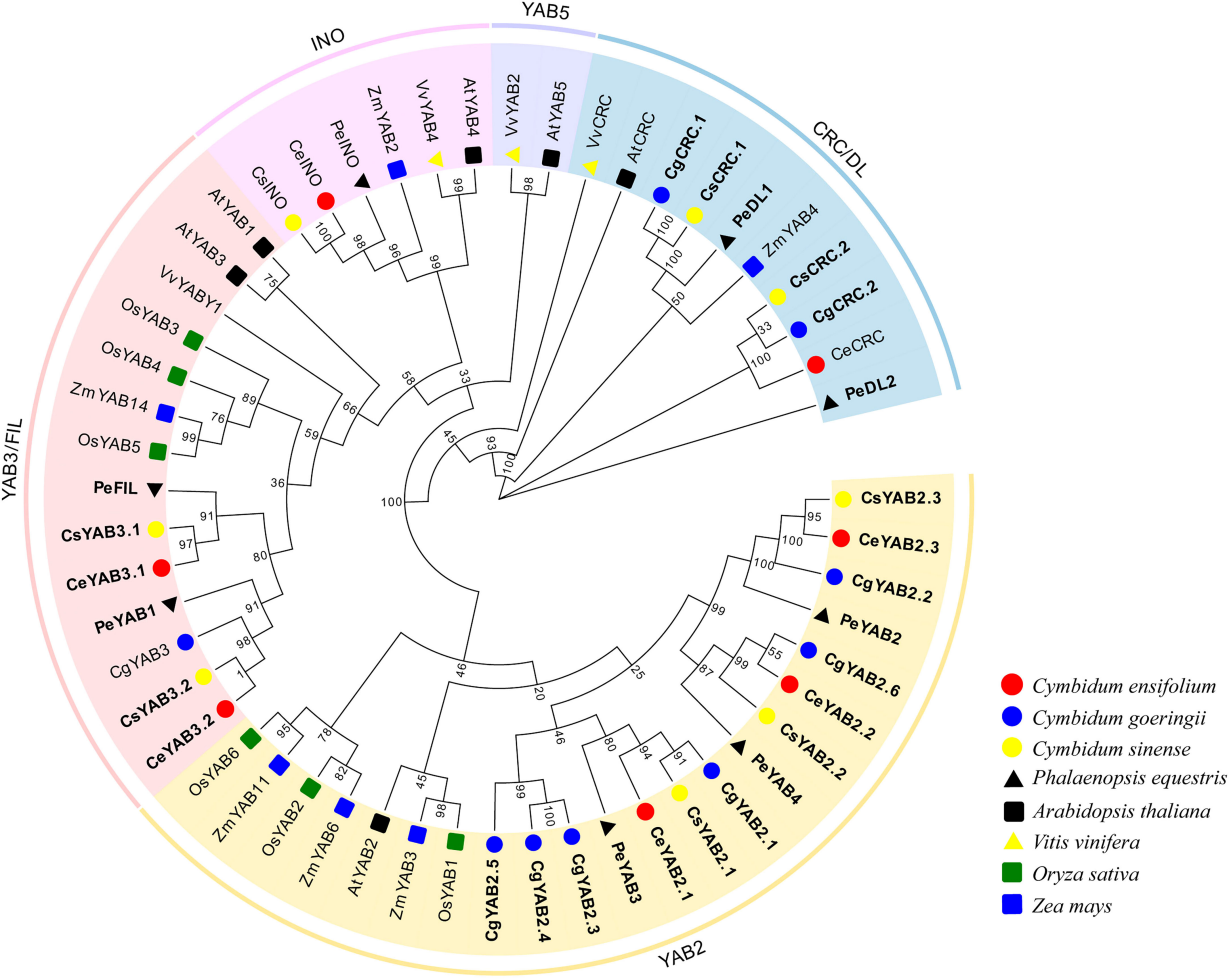
Phylogenetic tree of YABBY genes in eight plant species. The phylogenetic tree was created with the maximum-likelihood (ML) method using RAxML on the CIPRES Science Gateway web server (RAxML-HPC2 on XSEDE; Miller et al., 2011). Bootstrap values based on 1000 replicates are shown along the branches. Ce, *C. ensifolium*; Cg, *C. goeringii*; Cs, *C. sinensis*; At, *A. thaliana*; Os, *O. sativa*; Pe, *P. equestris*; Vv, *V. vinifera*; Zm, *Z. mays*; The duplicated genes are shown in bold.

To analyze the evolution patterns of YABBY genes in *Cymbidium* species, a phylogenetic tree was created by using the ML (maximum likelihood) method. Protein sequences from *C. ensifolium*, *C. goeringii*, *C. sinense*, *A. thaliana O. sativa*, *P. equestris*, *V. vinifera*, and *Z. mays* were used. The IDs of these species are listed in **Supplementary Table S3**. The results indicated that all *Cymbidium* species except *C. goeringii* have one member in the INO cluster. The number of YAB2 genes ranged from 3-6 (*C. ensifolium*: 3; *C. goeringii*: 6; *C. sinense*: 3). *C. goeringii* and *C. sinense* have two genes in the CRC subfamily, but *C. ensifolium* has only one. With the exception of *C. goeringii*, all *Cymbidium* species have two YAB3 genes (**Figure 1**).

### Domains and motifs of YABBY genes

**Figure 2:**
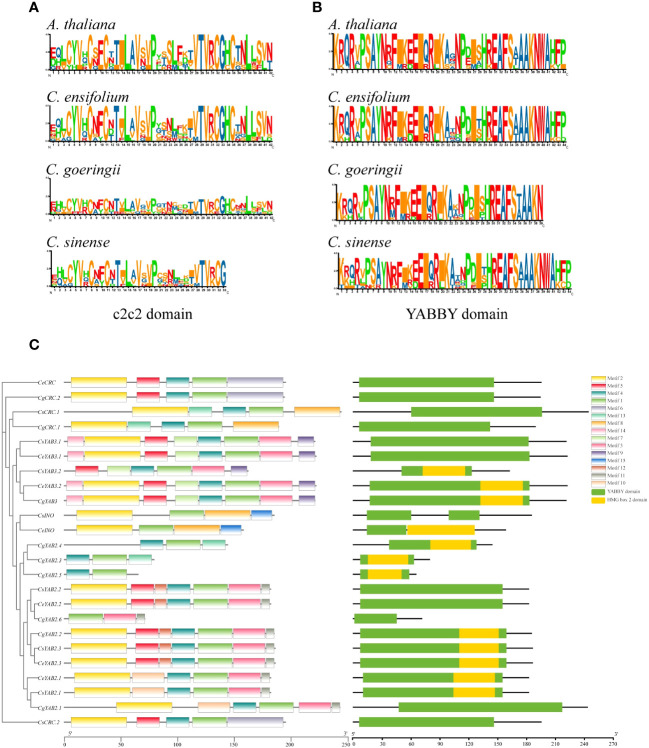
Conserved domains from three *Cymbidium* species and *A. thaliana*. **(A)** Sequence logo of the zinc-finger domain in the N-terminus. **(B)** Sequence logo of the YABBY domain in the C-terminus. **(C)** Motifs and conserved domains in the YABBY protein amino acid sequences in *Cymbidium* species.

To analyze the conserved domains of YABBY genes, the sequence logo of YABBY domains and c2c2 zinc-finger domains from three *Cymbidium* species and *A. thaliana* was generated. The multiple sequence alignment was also generated. The results showed that *Cymbidum* species and *A. thaliana* have highly conserved c2c2 zinc-finger domains and YABBY domains. However, the YABBY domain is more conserved than the c2c2 domain in *Cymbidium* species (**Figure 2**). Additionally, the motifs, domains, and phylogenetic tree of three *Cymbidium* species were analyzed (**Figure 2**). Fifteen motifs were analyzed by MEME software (**Supplementary Table S4**; Bailey et al., 2009). The results indicated that all the *Cymbidium* species have YABBY domains, and most YABBY genes of *Cymbidium* have motif 2 and motif 4. The findings also revealed that the conserved motifs of YABBY genes in the same clusters are similar.

### Chromosome distribution and collinear correlation analysis

**Figure 3:**
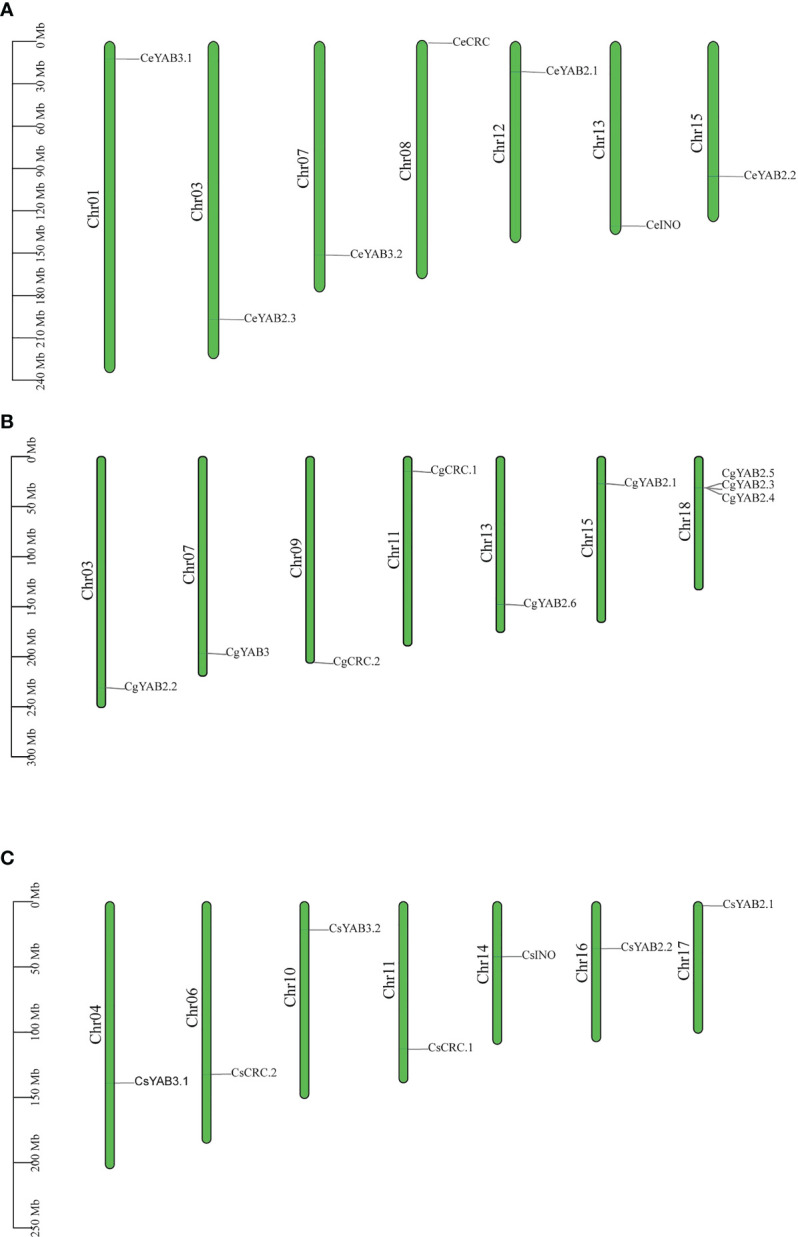
Chromosome distribution in three *Cymbidium* species. **(A)** Chromosome distribution in *C. ensifolium*. **(B)** Chromosome distribution in *C. goeringii*. **(C)** Chromosome distribution in *C. sinense*.

**Figure 4:**
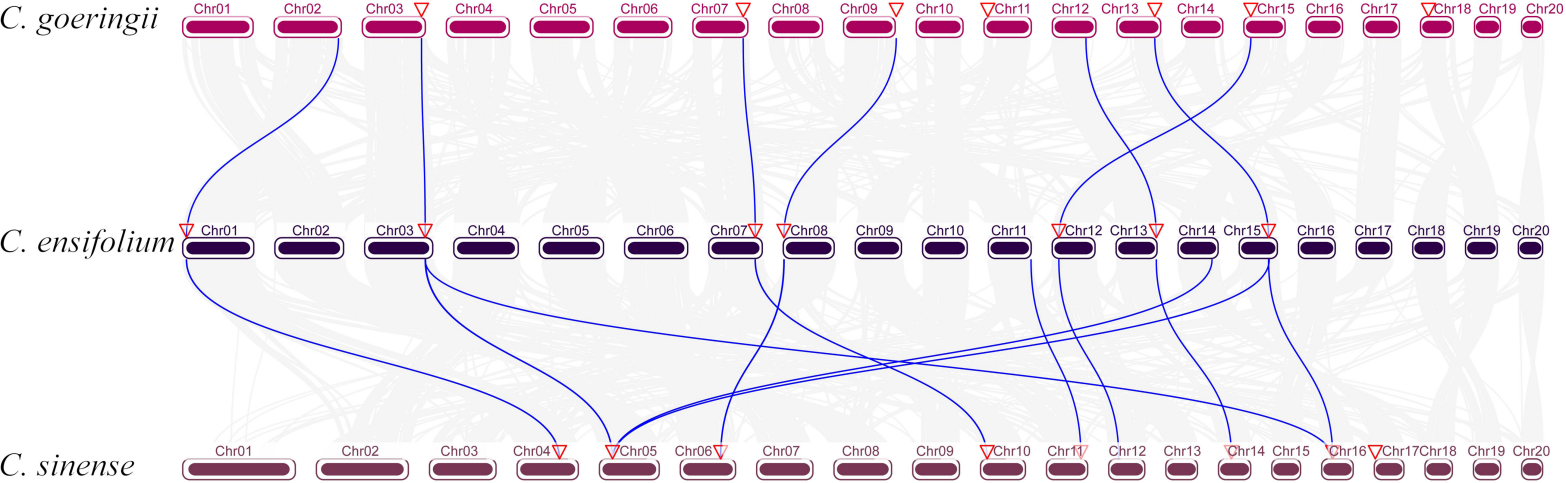
Collinear correlation analysis in three *Cymbidium* species.

To analyze the chromosome distribution of YABBY genes in three *Cymbidium* species, we create gene distribution maps. The results suggest that YABBY genes were distributed in seven chromosomes in *C. ensifolium*, *C. goeringii*, and *C. sinense* (**Figure 3**). In addition, YABBY genes were located in different chromosomes in *C. ensifolium* and *C. sinense.* Nevertheless, in *C. goeringii*, *CgYAB2.3*, *CgYAB2.4*, and *CgYAB2.5* were located on same chromosome (chr17). We also analyzed the syntenic relationships of YABBY genes in three *Cymbidium* species. There are seven, nine, and eight YABBY genes in *C. ensifolium*, *C. goeringii*, and *C. sinense* (**Figure 4**). The results indicated that almost all the YABBY genes displayed corresponding one-to-one relationships in these three *Cymbidium* species.

### *Cis*-element analysis of *C. ensifolium*, *C. goeringii*, and *C. sinense*

To predict the regulatory function of YABBY genes, we retrieved a 2000-bp region upstream of 24 YABBY genes and analyze them in *C. ensifolium*, *C. goeringii*, and *C. sinense*. We identified 12 types of *cis*-elements: abscisic acid responsiveness element, anaerobic induction element, auxin responsiveness element, circadian control element, defense and stress responsiveness element, endosperm expression element, light responsive element, low-temperature responsiveness element, MeJA-responsiveness element, meristem expression element, salicylic acid responsiveness element, and zein metabolism regulation element. In total, we found 412 *cis*-elements in three *Cymbidium* species, and *C. sinense* has most of the cis-elements (192/412), followed by *C. goeringii* (120/412), and *C. ensifolium* (100/412). The results also indicated that most of the elements were clustered in light-responsive elements (199/412), followed by MeJA-responsive elements (64/412), anaerobic induction element (27/412), and abscisic acid responsiveness element (24/412). All YABBY genes have light-responsive elements, and *CsYAB3.1* contains the most (35/199). In addition, only *CeYAB2.1*, *CeYAB3.2*, and *CgYAB2.1* have circadian control elements (**Figure S1**).

### Expression analysis of *C. ensifolium* and *C. goeringii*

**Figure 5:**
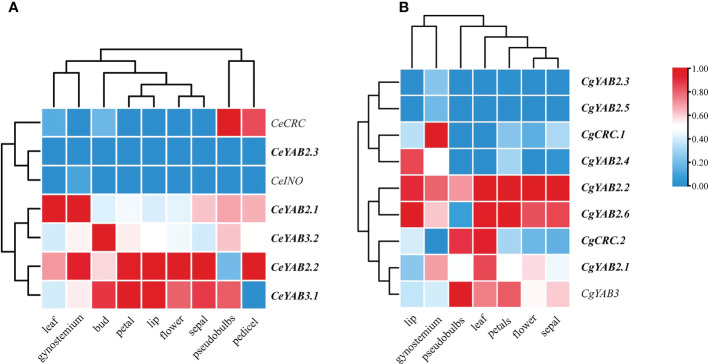
Expression patterns of YABBY genes in different organs from three *Cymbidium* species. **(A, B)** show the expression patterns of YABBY genes in different organs in *C. ensifolium* and *C. goeringii*. The heatmap was produced in Tbtools (Chen et al., 2018). The fragments per kilobase of transcript per million fragments (FPKM) values can be found in **Supplementary Table S5**. The duplicated genes are shown in bold.

To analyze the expression patterns of YABBY genes, we sampled vegetative and floral organs from *C. ensifolium* and *C. goeringii*. The results suggested that in *C. ensifolium*, *CeCRC* showed high expression in pseudobulbs and pedicel, *CeYAB2.1* and *CeYAB 2.2* showed high expression in leaf and gynostemium, and *CeYAB3.2* showed high expression in bud. *CeYAB2.1*, *CeYAB2.2*, *CeYAB3.1*, and *CeYAB3.2* had expression in both vegetative and floral organs (**Figure 5**). In *C. goeringii*, *CgCRC.1* showed high expression in gynostemium, and *CgCRC.2* showed high expression in pseudobulbs and gynostemium. *CgYAB3* showed high expression in pseudobulbs, leaves, and petals. *CgCRC.2*, *CgYAB2.1*, *CgYAB2.2*, *CgYAB2.6*, and *CgYAB3* had expression in both vegetative and floral organs.

### Expression patterns in leaves and three floral organs in *C. ensifolium*

**Figure 6:**
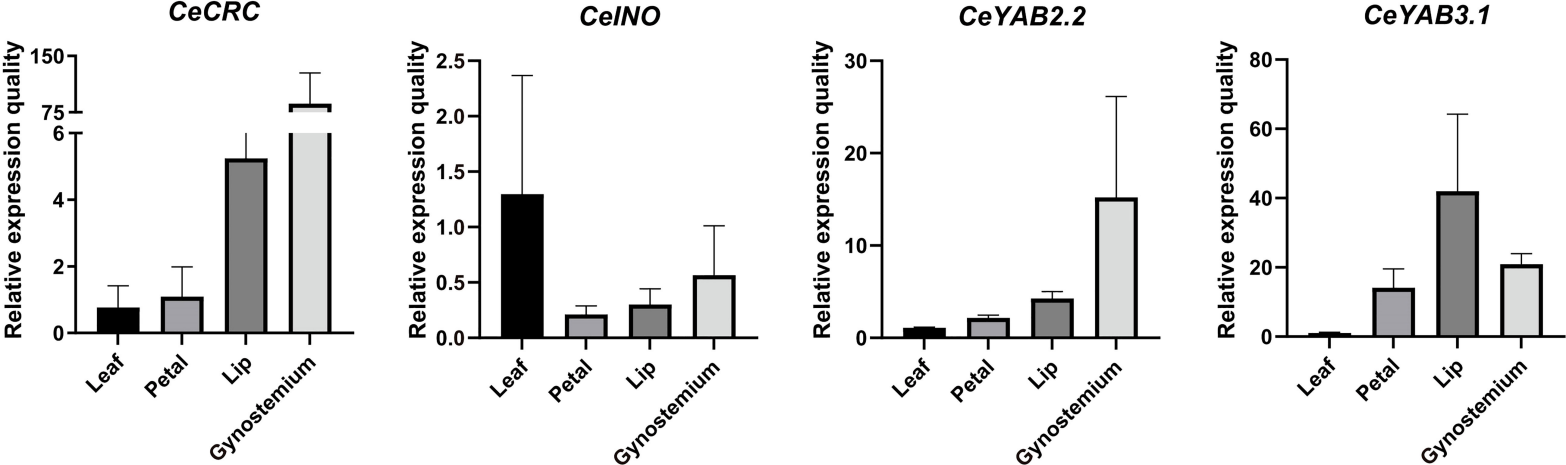
Relative expression patterns of YABBY genes in *C. ensifolium*. Raw data are listed in **Supplementary Tables S6** and **S7**.

To analyze the expression patterns of YABBY genes, we collected three floral organs (petal, lip, and gynostemium) and leaves from *C. ensifolium*. Four YABBY genes, *CeCRC*, *CeINO*, *CeYAB2.2*, and *CeYAB3.1* were chosen for RT–qPCR analysis. The results showed that *CeYAB3* showed high expression in the lip, petal, and gynostemium. *CeCRC* and *CeYAB2.2* showed high expression in gynostemium. *CeCRC*, *CeYAB2.2*, and *CeYAB3.1* had higher expression levels in floral organs than in leaves. However, the expression levels in leaves were higher than those in floral organs from *CeINO* (**Figure 6**).

## Discussion

YABBY genes, which include a zinc finger domain near the N-terminus and a helix-loop-helix domain (YABBY domain) near the C-terminus, play important roles in lamina development in cotyledons, floral organs, and outer ovule integuments (Finet et al., 2016). In monocots, eight genes have been identified in *O. sativa*; in core eudicots, six YABBY genes have been found in *A. thaliana* (Bowman and Smyth, 1999; Sawa et al., 1999; Villanueva et al., 1999). Orchidaceae, belonging to monocots, is one of the largest angiosperm families and show unique flower morphologies and reproductive biology (Hsiao et al., 2011; Christenhusz and Byng, 2016). Recent studies have indicated that six YABBY genes were identified in *A. shenzhenica*, eight in *D. catenatum*, five in *G. elata*, and eight in *P. equestris* (Chen et al., 2020). However, studies of YABBY genes in *Cymbidium* are still limited. In this study, YABBY genes were identified in three *Cymbidium* species and the number of YABBY genes ranged from 7-9 (*C. ensifolium*: 7; *C. goeringii*: 9; *C. sinense*: 8). These results indicated that the number of YABBY genes in *Cymbidum* orchids were comparable to those in monocot and dicot species. However, the absence of YABBY genes in YAB 5 subfamily in orchids and other monocots is an exception.

The phylogenetic analysis indicated that YABBY genes in *Cymbidium* species are clustered into four subfamilies: YAB2, CRC, YAB3, and INO. There were no YABBY genes that clustered in the YAB5 subfamily. The results were consistent with some monocot species, such as *A. shenzhenica*, *D. catenatum*, *G. elata*, pineapple, and rice (Toriba et al., 2007; Li et al., 2019; Chen et al., 2020). However, seven species of magnoliids and *A. thaliana* have YABBY genes clustered in the YAB 5 clade (Siegfried et al., 1999; Liu et al., 2021). Early in the evolution of angiosperms, the lineages of basal flowering plants diverged, and then the magnoliids, eudicots, and monocots underwent rapid diversification (Tang et al., 2014; Chen et al., 2019). Magnoliids have two cotyledons and pollen with a single pore, and they are not monocots or eudicots (Tang et al., 2014). Recent reports also studied the comparative development of the androecial form in the Zingiberales and found one YAB2 gene, which was less homologous to YAB5 (De Almeida et al., 2014). Based on this, they suggested that after the divergence of monocots and eudicots, duplication led to separate YAB2 and YAB5 gene lineages (De Almeida et al., 2014). The YAB5 clade was exclusively composed of basal angiosperms and eudicot in recent studies (Chen et al., 2017; Liu et al., 2021). These results suggested that YAB5 gene clade might have been lost in monocot plants.

INO are restricted to the development of the outer ovule integument (Villanueva et al., 1999). Interestingly, we found *C. ensifolium* and *C. sinense* only has one number in the INO clade. These results were consistent with *A. shenzhenica*, *A. thaliana*, *D. catenatum*, *G. elata*, *P. equestris*, and *V. vinifera*, and indicated INO clade genes might be conserved in angiosperm plants and play essential roles in the outer integument (Siegfried et al., 1999; Zhang et al., 2019; Chen et al., 2020).

YABBY genes include a zinc finger domain near the N-terminus and a helix-loop-helix domain (YABBY domain) near the C-terminus. The results showed that the YABBY domain is more conserved than the c2c2 domain in three *Cymbidium* species. Fifteen motifs were analyzed in three *Cymbidum* species, and most YABBY genes of *Cymbidium* have motif 4 and motif 2. These findings revealed that the gene structure of YABBY genes are conserved during evolution. In the evolution of gene families, two main methods are tandem duplication and fragment duplication (Cannon et al., 2004). Chromosomal location analysis results suggested that YABBY genes were located in different chromosomes in *C. ensifolium* and *C. sinense.* But in *C. goeringii*, *CgYAB2.3*, *CgYAB2.4*, and *CgYAB2.5* were located on same chromosome (chr17). The results indicated those genes might be tandem repeat genes. The syntenic relationships analysis indicated that almost every YABBY gene displayed corresponding one-to-one relationships in these three *Cymbidium* species.

*Cis*-elements were found in promoter areas in YABBY genes. The results indicated that most of the elements were clustered in light-responsive elements (199/412), followed by MeJA-responsive elements (64/412), anaerobic induction elements (27/412), and abscisic acid responsiveness element (24/412). The MeJA (methyl jasmonate) is a phytohormone involved in defense signaling of plants (Howe, 2004). The results indicated YABBY genes might play essential roles in plant growth and stress.

The growth of lateral organs in *A. thaliana* is thought to be redundantly controlled by the genes YAB2 and FIL, which are expressed in the leaves, cotyledons, and floral organs (Siegfried et al., 1999; Rudall and Bateman, 2002). FIL gene orthologues have similarly acted in flower development in *Oryza* (Tanaka et al., 2017). Our study indicated that three *Cymbidium* species contained one or two FIL genes and had high expression in the floral organs of *C. ensifolium* and *C. goeringii*. The results suggested that FIL may play important roles in the development of floral organ in *Cymbidium* species. CRC showed high expression in pseudobulbs in *C. ensifolium* and *C. goeringii*, and CRC showed high expression in pedicels in *C. ensifolium*. CRC also showed high expression in gynostemium in *C. goeringii*. The results suggested that CRC in different *Cymbidum* had different expression patterns. INO expressed in the gynostemium and pedicel in *C. ensifolium*. It may play important roles in the development of gynostemium and pedicel. YAB2 genes (*CeYAB2.1*, *CeYAB2.2*, *CgYAB2.1*, *CgYAB2.2*, and *CgYAB2.6*) showed high expression in all organs in *Cymbidium* species, indicating that the YAB2 clade may have functions in both reproductive and vegetative organs.

The results indicated that YABBY genes in *Cymbidium* species showed higher expression in reproductive tissues than in vegetative tissues. The results were consistent with the expression patterns reported in *A. shenzhenica*, *D. catanum*, and *P. equestris* (Chen et al., 2020). RT–qPCR analysis showed that *CeCRC*, *CeYAB2.2*, and *CeYAB3.1* have higher expression levels in floral organs than in leaves. However, the expression levels in leaves were slightly higher than those in floral organs in *CeINO.* These findings indicated that YABBY genes play important roles in floral organ development in orchids. Orchids display unique flower morphologies, and their flowers possess several reliable floral morphological synapomorphies, including a gynostemium (a fused structure of the pistils and stamens) (Chase et al., 2003; Tsai et al., 2004). The results of this study indicated that *CeCRC* might play essential roles in floral organs, especially in gynostemium.

## Data availability statement

The data presented in the study are deposited in the National Centre for Biotechnology Information (NCBI) and National Genomics Data Center (NGDC). The raw data can be found under the following accession numbers: SAMN20059972 (NCBI), PRJNA749652 (NCBI) and PRJCA005355 (NGDC).

## Author contributions

SL, Z-JL, and DZ contributed to conceptualization and validation. Q-QW, Y-YL, and ZZ prepared the original draft. Q-QW, JC, and M-JZ analyzed data, Q-QW and XL make the images. All authors contributed to the article and approved the submitted version.

## Funding

This research was funded by Forestry Peak Discipline Construction Project of Fujian Agriculture and Forestry University (72202200205), The National Natural Science Foundation of China (no. 31870199), The Key Laboratory of National Forestry and Grassland Administration for Orchid Conservation and Utilization Construction Funds (Grant 115/118990050, 115/KJG18016A), Natural Science Foundation of Zhejiang Province (Grant nos. LY20C160005, LY19C150003), Key Research and Development Program of Zhejiang Province (Grant no. 2021C02043), and Wenzhou Agricultural New Variety Breeding Cooperative Group Project (Grant no. 2019ZX004-3).

## Conflict of interest

The authors declare that the research was conducted in the absence of any commercial or financial relationships that could be construed as a potential conflict of interest.

## Publisher’s note

All claims expressed in this article are solely those of the authors and do not necessarily represent those of their affiliated organizations, or those of the publisher, the editors and the reviewers. Any product that may be evaluated in this article, or claim that may be made by its manufacturer, is not guaranteed or endorsed by the publisher.
