# Supplementary material for: Genome-wide identification of YABBY genes in three Cymbidium species and expression patterns in C. ensifolium (Orchidaceae)
Source: Front Plant Sci. 2022 Nov 24;13:995734. doi: 10.3389/fpls.2022.995734 (PMC9729879; doi:10.3389/fpls.2022.995734)
Supplement: Supplementary file 1 [file DataSheet_1.docx]

***Supplementary Material***

**Genome-wide identification of YABBY genes in three *Cymbidium* species and expression patterns in *C. ensifolium* (Orchidaceae)**

Qian-Qian Wang^1^, Yuan-Yuan Li^1^, Jiating Chen^1^, Meng-Jia Zhu ^2^, Xuedie Liu^1^, Zhuang Zhou^1,3^, Diyang Zhang^1^, Zhong-Jian Liu^1,2,3*^ and Siren Lan^1,2*^

^1^ Key Laboratory of National Forestry and Grassland Administration for Orchid Conservation and Utilization at College of Landscape Architecture, Fujian Agriculture and Forestry University, Fuzhou 350002, China.

^2^ College of Forestry, Fujian Agriculture and Forestry University, Fuzhou, China,

^3^ Zhejiang Institute of Subtropical Crops, Zhejiang Academy of Agricultural Sciences, Wenzhou 325005, China.

*** Correspondence:**

Corresponding Authors

Z.-J. L (zjliu@fafu.edu.cn), S. L. (lkzx@fafu.edu.cn).

**SUPPLEMENTARY TABLE LEGENDS**

**TABLE S1|** Subcellular localization predicted by Plant-mPLoc;

**TABLE S2|** Secondary structure of YABBY proteins.

**TABLE S3|** Proteins sequences IDs used in the phylogenetic tree

**TABLE S4|** Conserved motifs in YABBY proteins using MEME website.

**FIGURE S1 |** Promoter analysis of YABBY genes in three *Cymbidium* species*.*

**TABLE S5|** The FPKM values of YABBY genes in *C. ensifolium* and *C. sinense*.

**TABLE S6|** The expression values of YABBY genes in *C. ensifolium*.

**TABLE S7|** RT-qPCR primers in *C. ensifolium*.

**TABLE S1|** Subcellular localization predicted by Atsubp

| **Gene ID^1^** | **Name** | **Localization** |
| --- | --- | --- |
| JL015423 | CeCRC | Nucleus |
| JL011339 | CeYAB2.1 | Nucleus |
| JL000262 | CeYAB2.2 | Nucleus |
| JL008521 | CeYAB3.1 | Nucleus |
| JL005041 | CeYAB3.2 | Nucleus |
| JL005324 | CeYAB2.3 | Nucleus |
| JL012731 | CeINO | Nucleus |
| GL09549 | CgCRC.1 | Nucleus |
| GL08212 | CgCRC.2 | Nucleus |
| GL09374 | CgYAB3 | Nucleus |
| GL12804 | CgYAB2.1 | Nucleus |
| GL19435 | CgYAB2.2 | Nucleus |
| GL30075 | CgYAB2.3 | Nucleus |
| GL30077 | CgYAB2.4 | Nucleus |
| GL30076 | CgYAB2.5 | Nucleus |
| GL10103 | CgYAB2.6 | Nucleus |
| Mol018025 | CsCRC.1 | Nucleus |
| Mol010228 | CsCRC.2 | Nucleus |
| Mol006632 | CsYAB2.1 | Nucleus |
| Mol000581 | CsYAB2.2 | Nucleus |
| Mol007225 | CsYAB3.1 | Nucleus |
| Mol011195 | CsYAB2.3 | Nucleus |
| Mol003404 | CsYAB3.2 | Nucleus |
| Mol004846 | CsINO | Nucleus |

**TABLE S2|** Secondary structure of YABBY proteins.

| **Gene ID^1^** | **Name** | Alpha helix | Extended strand | Beta turn | Random coil |
| --- | --- | --- | --- | --- | --- |
| JL015423 | CeCRC | 21.65% | 19.59% | 6.70% | 52.06% |
| JL011339 | CeYAB2.1 | 24.86% | 17.13% | 5.52% | 52.49% |
| JL000262 | CeYAB2.2 | 16.57% | 18.23% | 3.87% | 61.33% |
| JL008521 | CeYAB3.1 | 28.51% | 12.22% | 3.62% | 55.66% |
| JL005041 | CeYAB3.2 | 23.53% | 13.12% | 3.17% | 60.18% |
| JL005324 | CeYAB2.3 | 21.62% | 14.59% | 4.32% | 59.46% |
| JL012731 | CeINO | 32.48% | 17.20% | 5.10% | 45.22% |
| GL09549 | CgCRC.1 | 21.69% | 16.93% | 6.35% | 55.03% |
| GL08212 | CgCRC.2 | 21.65% | 19.59% | 6.70% | 52.06% |
| GL09374 | CgYAB3 | 23.53% | 14.03% | 3.62% | 58.82% |
| GL12804 | CgYAB2.1 | 27.98% | 14.81% | 5.35% | 51.85% |
| GL19435 | CgYAB2.2 | 20.54% | 16.22% | 6.49% | 56.76% |
| GL30075 | CgYAB2.3 | 40.51% | 2.53% | 8.86% | 48.10% |
| GL30077 | CgYAB2.4 | 38.19% | 9.72% | 11.11% | 40.97% |
| GL30076 | CgYAB2.5 | 44.62% | 3.08% | 9.23% | 43.08% |
| GL10103 | CgYAB2.6 | 52.11% | 4.23% | 2.82% | 40.85% |
| Mol018025 | CsCRC.1 | 25.51% | 17.28% | 7.00% | 50.21% |
| Mol010228 | CsCRC.2 | 21.65% | 19.59% | 6.70% | 52.06% |
| Mol006632 | CsYAB2.1 | 22.65% | 16.57% | 4.97% | 55.80% |
| Mol000581 | CsYAB2.2 | 19.34% | 21.55% | 7.73% | 51.38% |
| Mol007225 | CsYAB3.1 | 21.82% | 12.73% | 4.09% | 61.36% |
| Mol011195 | CsYAB2.3 | 21.62% | 14.59% | 4.32% | 59.46% |
| Mol003404 | CsYAB3.2 | 34.78% | 6.21% | 3.11% | 55.90% |
| Mol004846 | CsINO | 35.33% | 17.39% | 4.89% | 42.39% |
|  | Average | 27.61% | 14.13% | 5.65% | 52.60% |

**TABLE S3.** Proteins sequences IDs used in phylogenetic tree.

| Species | Protein name | IDs |
| --- | --- | --- |
| *Oryza sativa* | CRC/DL | AY494713 |
| *Oryza sativa* | INO | LOC9268430 |
| *Oryza sativa* | FIL | Q01JG2 |
| *Oryza sativa* | YAB2 | Q10FZ7 |
| *Oryza sativa* | YAB2 | Q2QM17 |
| *Oryza sativa* | FIL | Q6H668 |
| *Oryza sativa* | YAB2 | Q7XIM7 |
| *Oryza sativa* | FIL | Q8L556 |
| *Vitis vinifera* | FIL | XP_002266233 |
| *Vitis vinifera* | CRC/DL | XP_010650015 |
| *Vitis vinifera* | FIL | XP_010661657 |
| *Zea mays* | YAB2 | ACG42776 |
| *Zea mays* | FIL | BT040048 |
| *Zea mays* | FIL | NP_001105230 |
| *Zea mays* | YAB2 | NP_001140845 |
| *Zea mays* | YAB2 | NP_001141765 |
| *Zea mays* | INO | NP_001149494 |
| *Zea mays* | CRC/DL | NP_001183591.1 |
| *Zea mays* | FIL | KJ727991 |
| *Apostasia shenzhenica* | FIL | PKA48429 |
| *Apostasia shenzhenica* | CRC/DL | PKA49723 |
| *Apostasia shenzhenica* | YAB2 | PKA56987 |
| *Apostasia shenzhenica* | YAB2 | PKA62133 |
| *Apostasia shenzhenica* | FIL | PKA64175 |
| *Dendrobium catenatum* | YAB2 | PKU64994 |
| *Dendrobium catenatum* | CRC/DL | PKU69929 |
| *Dendrobium catenatum* | FIL | PKU79106 |
| *Dendrobium catenatum* | CRC/DL | PKU86051 |
| *Dendrobium catenatum* | FIL | XP_020674395 |
| *Dendrobium catenatum* | CRC/DL | XP_020678472 |
| *Dendrobium catenatum* | YAB2 | XP_020682129 |
| *Phalaenopsis equestris* | CRC/DL | XP_020572967 |
| *Phalaenopsis equestris* | YAB2 | XP_020574278 |
| *Phalaenopsis equestris* | CRC/DL | XP_020583837 |
| *Phalaenopsis equestris* | INO | XP_020589671 |
| *Phalaenopsis equestris* | FIL | XP_020592548 |
| *Phalaenopsis equestris* | FIL | XP_020593943 |
| *Phalaenopsis equestris* | YAB2 | XP_020596475 |

**TABLE S4.** Conserved motifs in YABBY proteins using MEME website.

| Motif | Sequences | E-value | Sites | Width |
| --- | --- | --- | --- | --- |
| 1 | YNRFIKEEIQRIKATNPDISHREAFSAAAKN | 7.0e-582 | 24 | 31 |
| 2 | SPEHLCYVHCNFCNTVLAVSVPCSNLLKTVTVRCGHCTNLLSVNMGGLLQ | 8.7e-560 | 19 | 50 |
| 3 | WAHFPHIHFGLNHDSSLQVKIDDPITAZE | 1.7e-223 | 14 | 29 |
| 4 | EEENQQRILPNRPPEKRQRVP | 2.6e-170 | 21 | 21 |
| 5 | NQLHQGHNFLSEVQHNLLESS | 2.4e-063 | 13 | 21 |
| 6 | WAKCDPRALFYSASSTSGTRRATTTIQQEKRSGAPVEGFDVSKHGQLHRM | 9.0e-062 | 3 | 50 |
| 7 | MLRETQPCLSNNSMPPLMKGK | 1.3e-034 | 5 | 21 |
| 8 | WAVFPRFDNKEPSEGCSTRREGGKGRDDEAFDQFDZREVQR | 2.4e-028 | 4 | 41 |
| 9 | GFFASAAANMEVAPF | 1.3e-024 | 5 | 15 |
| 10 | DTLQNTQYGVKSQDLHLHSGPISGCSRTV | 1.9e-022 | 3 | 29 |
| 11 | GQESQGYY | 5.1e-020 | 9 | 8 |
| 12 | SRCNRIPLFNA | 2.3e-010 | 5 | 11 |
| 13 | AHNSPQPGGFQDPCGDVRKGZ | 1.2e-008 | 4 | 21 |
| 14 | SSSAAAFSLEQJSPP | 5.1e-006 | 4 | 15 |
| 15 | FQERKALRHAILMDTDALA | 2.1e-004 | 2 | 19 |

**FIGURE S1 |** Promoter analysis of YABBY genes in three *Cymbidium* species.


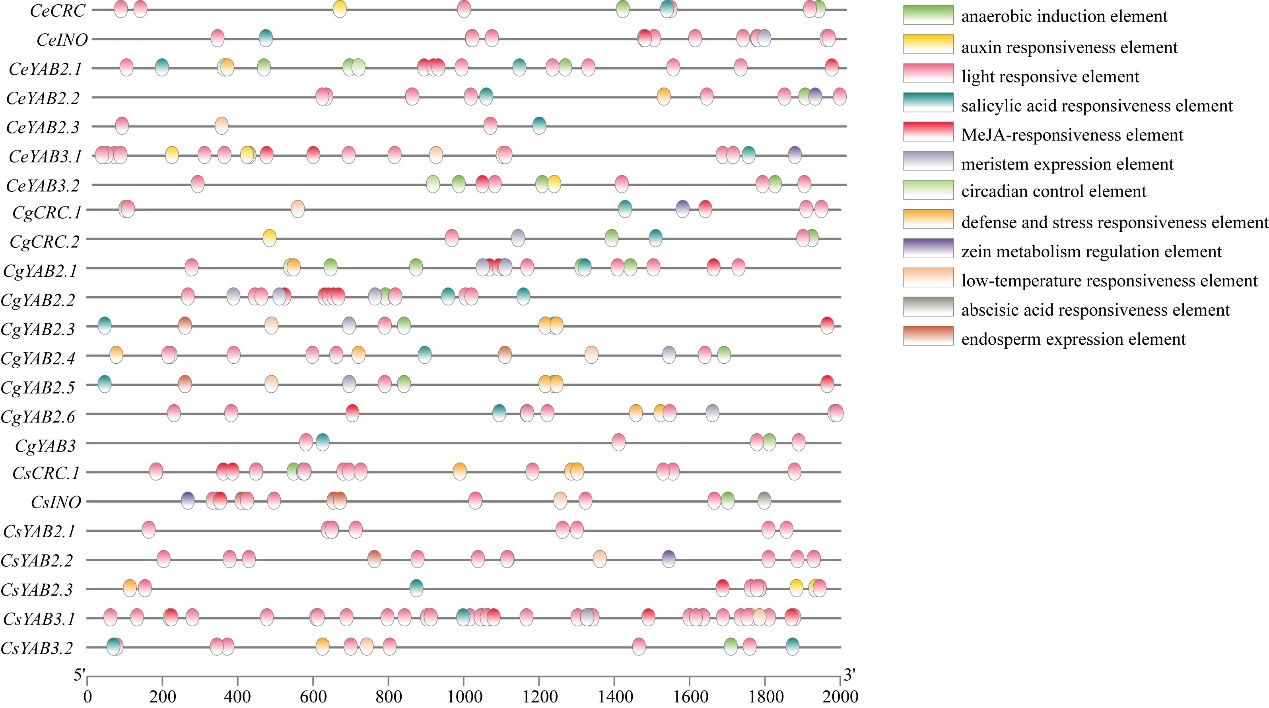


**TABLE S5|** The FPKM values of YABBY genes in *C. ensifolium* and *C. goeringii*.

| Gene ID | | Le | | Ps | | Pe | B | | F | | Se | | Pe | | Li | | Gs |
| --- | --- | --- | --- | --- | --- | --- | --- | --- | --- | --- | --- | --- | --- | --- | --- | --- | --- |
| *CeCRC* | | 1 | | 34 | | 202 | 2 | | 0 | | 0 | | 0 | | 0 | | 0 |
| *CeYAB2.1* | | 186 | | 10 | | 77 | 13 | | 11 | | 25 | | 10 | | 6 | | 766 |
| *CeYAB2.2* | | 38 | | 1 | | 443 | 39 | | 347 | | 194 | | 173 | | 114 | | 510 |
| *CeYAB2.3* | | 0 | | 0 | | 2 | 0 | | 0 | | 0 | | 0 | | 0 | | 0 |
| *CeYAB3.1* | | 7 | | 17 | | 2 | 261 | | 108 | | 100 | | 194 | | 103 | | 38 |
| *CeYAB3.2* | | 7 | | 8 | | 39 | 549 | | 13 | | 7 | | 17 | | 10 | | 35 |
| *CeINO* | | 0 | | 0 | | 2 | 0 | | 0 | | 0 | | 0 | | 0 | | 1 |
| Gene ID | Le | | Ps | | F | | | Se | | Pe | | Li | | Gs | |  | |
| *CgCRC.1* | 0 | | 0 | | 0.86 | | | 3.12 | | 1.75 | | 2.42 | | 198.45 | |  | |
| *CgCRC.2* | 48.76 | | 13.65 | | 1.16 | | | 0.99 | | 2.71 | | 3.16 | | 0.11 | |  | |
| *CgYAB2.1* | 33 | | 3.18 | | 11.36 | | | 6.29 | | 7.09 | | 1.39 | | 37.6 | |  | |
| *CgYAB2.2* | 63.18 | | 7.37 | | 76.34 | | | 85.49 | | 74.07 | | 24.07 | | 68.63 | |  | |
| *CgYAB2.3* | 0 | | 0 | | 0 | | | 0 | | 0.05 | | 0 | | 2.83 | |  | |
| *CgYAB2.4* | 0 | | 0 | | 0 | | | 0.13 | | 2.64 | | 19.81 | | 12.85 | |  | |
| *CgYAB2.5* | 0 | | 0 | | 0 | | | 0 | | 0 | | 0 | | 1.93 | |  | |
| *CgYAB2.6* | 54.03 | | 0.26 | | 36.1 | | | 44 | | 64.39 | | 34.04 | | 26.52 | |  | |
| *CgYAB3* | 20.95 | | 19.61 | | 9.05 | | | 13.91 | | 32.85 | | 2.65 | | 7.78 | |  | |

The tissues were bud (B), flower (F), leaves (Le), pseudobulbs (Ps), petals (Pe), sepals (Se), lip (Li), gynostemium (Gs), Pedicel (Ped).

**TABLE S6|** The expression values of YABBY genes in *C. ensifolium*.

|  | Le1 | Le2 | | Le3 | | Pe1 | | Pe2 | | Pe3 | | Lip1 | | Lip2 | | Lip3 | | gyno1 | | gyno2 | | gyno3 | |  |
| --- | --- | --- | --- | --- | --- | --- | --- | --- | --- | --- | --- | --- | --- | --- | --- | --- | --- | --- | --- | --- | --- | --- | --- | --- |
| *CeCRC* | 0.309 | | 1.226 | | NO | | 2.062 | | 0.312 | | 0.903 | | 5.932 | | 4.553 | | NO | | 121.856 | | 95.986 | | 41.327 | |
| *CeINO* | 0.581 | | 0.780 | | 2.528 | | 0.236 | | 0.125 | | 0.272 | | 0.202 | | 0.236 | | 0.463 | | 0.354 | | 0.267 | | 1.078 | |
| *CeYAB2.2* | 1.116 | | 1.016 | | 1.152 | | 2.501 | | 1.933 | | 2.028 | | 4.985 | | 4.310 | | 3.459 | | 8.683 | | 9.107 | | 27.816 | |
| *CeYAB 3.1* | 0.954 | | 0.813 | | 1.289 | | 14.283 | | 19.514 | | 8.571 | | 58.701 | | 50.505 | | 16.772 | | 24.154 | | 20.493 | | 17.978 | |

Le, Pe, Lip, gyno indicate leaf, petal, lip, and gynostemium in *C. ensifolium*.

**TABLE S7|** RT-qPCR primers in *C. ensifolium*.

| Gene |  | Primer |
| --- | --- | --- |
| *CeGADPH* | F | GCTACGTTGATGAGGATCTTGTG |
| *(**JL008987)* | R | GTTGGCAATGTGACGGATGA |
| *CeCRC* | F | CCACACCGCGAAGCATTTAG |
|  | R | TGGTTGTTGTTGCCCTCCTT |
| *CeINO* | F | TACAGTGAAATGCGGGCA |
|  | R | TGTGGGTGATGCTTGGAT |
| *CeYAB2.2* | F | CCTCAGTCAATTCCTCCTCAAG |
|  | R | TTGTGGATGTAGATCAGGCATATC |
| *CeYAB3.1* | F | TCTTGGAGGAAGTGCTGAGT |
|  | R | CGGTTGTATGCTGATGGAACT |

The RT-qPCR primer of YABBY genes in *C. ensifolium* were designed by the Primer Premier 5 software. The *C. ensifolium* *Glyceraldehyde-3-phosphate dehydrogenase* (GAPDH) was obtained from its genome data. F, forward; R, reverse.
